# Predictors of Nephrostomy Catheter Dislodgement: Insights from a Retrospective Analysis

**DOI:** 10.1007/s00270-025-04020-y

**Published:** 2025-04-09

**Authors:** Ali Dablan, Zöhre Okur, Mehmet Cingöz, Çağrı Erdim, Mustafa Fatih Arslan, Oğuzhan Türksayar, Hamit Özgül, Tevfik Güzelbey, İlhan Nahit Mutlu

**Affiliations:** Department of Radiology, Basaksehir Cam and Sakura City Hospital, 34488 Istanbul, Turkey

**Keywords:** Nephrostomy, Percutaneous, Catheter dislodgement, Risk Factors

## Abstract

**Purpose:**

To identify the anatomical and technical factors associated with unintended nephrostomy catheter dislodgement (NCD).

**Materials and Methods:**

A retrospective review of 742 percutaneous nephrostomy (PCN) procedures carried out between June 2020 and June 2024 was conducted. Thirty-eight patients with spontaneous NCD were assigned to the dislodgement group, and 38 matched controls were selected using propensity score matching.. Key measurements included cortex-to-skin distance, paravertebral muscle area, psoas muscle area, subcutaneous fat thickness, and renal parenchymal thickness.

**Results:**

Patients with NCD were similar in age and sex. No significant differences were observed in subcutaneous fat thickness, muscle thickness, or renal parenchymal thickness between the groups. However, cortex-to-skin distance was significantly shorter in the NCD group (*p* = 0.001). ROC analysis identified an optimal threshold of 46.65 mm for cortex-to-skin distance, with a sensitivity of 92.1%, specificity of 39.5%, and a positive predictive value of 60.3% (AUC = 0.67).

**Conclusion:**

Shorter cortex-to-skin distance is a key predictor of NCD. Patients with shorter cortex-to-skin distances, may benefit from closer monitoring and targeted preventive measures to reduce the risk of dislodgement.

**Graphical Abstract:**

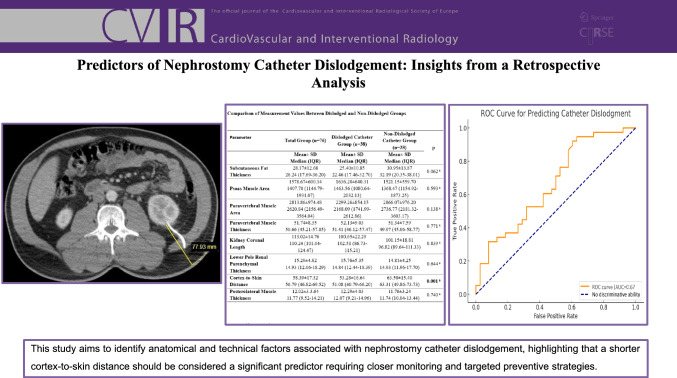

## Introduction

Urinary obstructions, whether due to functional, anatomical, or pathological causes, can lead to significant complications in the renal system. These obstructions increase pressure within the collecting system, potentially causing nephron loss and renal atrophy if untreated. Common etiologies include renal stones and malignancies, necessitating prompt intervention to preserve renal function and prevent further damage [1].

Percutaneous nephrostomy (PCN) is a minimally invasive procedure to alleviate urinary obstructions, particularly in cases of urinary retention or when urinary diversion is required. This procedure involves catheter insertion into the renal collecting system under imaging guidance (ultrasound and/or fluoroscopy), ensuring effective urine drainage. PCN is widely performed and critical in protecting renal function and preventing nephron loss [2–7].

PCN aims to prevent further urinary system damage until the underlying obstruction is resolved. Nephrostomy catheters are periodically replaced to prevent catheter-related complications, such as pyelonephritis, and maintain patency. Replacement intervals vary, typically ranging from 6 weeks to 3 months [8–10].

Despite its benefits, PCN is associated with procedural complications (e.g., bleeding, sepsis, urine leakage) and catheter-related issues (e.g., obstruction, dislodgment). Catheter dislodgment is among the most frequent complications, with reported incidence rates ranging from 1 to 37.6% [3–6, 8, 11, 12]. Dislodgment increases infection and bleeding risks, often necessitating additional interventions, contributing to higher healthcare costs, and increasing the workload for providers [8, 13, 14].

Although external fixation devices, sutures, and regular catheter checks reduce dislodgment risks, limited data exist on predictors of catheter dislodgment [8, 15, 16]. This study aimed to investigate the etiology of unintended nephrostomy catheter dislodgment (NCD) and identify associated technical and anatomical factors.

## Materials and Methods

The current retrospective study was approved by the institutional review board (IRB), which waived the requirement for informed consent for study participation due to its retrospective design. However, informed consent was obtained from all patients prior to the procedure as part of standard clinical practice.

### Patient Cohort

A retrospective review of 742 PCN procedures performed between June 2020 and June 2024 was conducted. Thirty-eight patients with documented evidence of spontaneous NCD formed the dislodgement group, and 38 patients without NCD served as the control group.

Control patients were selected using a 1:1 nearest-neighbor matching approach based on propensity scores. Age and sex were chosen as matching variables due to their clinical relevance and availability in the dataset. Age correlates with anatomical and physiological changes, such as reduced tissue integrity, which may influence catheter stability. Similarly, sex differences, such as muscle mass and fat distribution, could impact catheter dislodgment risks. These variables were normalized using Min–Max normalization to ensure equal weighting in propensity score calculations. Propensity scores were calculated via logistic regression, and each dislodgement group patient was matched to a control patient with the closest score. If no exact match was found, up to four nearest neighbors were considered.

Exclusion Criteria: Patients with prophylactically placed nephrostomy catheters, early post-procedure removal, infection, abscess, or hematoma in the nephrostomy tract, bedridden status, accidental trauma, or inappropriate mental status were excluded. Patients without a non-contrast CT scan within 3 months post-procedure or with a history of surgical intervention or radiotherapy involving the kidney were also excluded. Cases with imaging artifacts or renal axis anomalies were excluded.

### Percutaneous Nephrostomy Procedure and Patient Evaluation

Six different interventional radiologists, each with a minimum of 3 years of experience, carried out the PCN catheter placement. The procedure was carried out under ultrasound guidance, using an 18G needle to access the lower pole calyces. An 8F pigtail nephrostomy catheter (Argon Medical Devices, Plano, TX, USA) was placed after imaging with fluoroscopy. The catheter was secured to the skin using a non-absorbable 2/0 Silk surgical suture and tied with multiple knots in a braided pattern by the interventional radiologist who performed the procedure. Non-contrast abdominal CT scans were carried out using a 16-slice CT scanner (Somatom, Siemens, Germany). The evaluation of all non-contrast abdominal CT scans was conducted on a workstation using Fonet PACS software (Fonet Information Technologies, Ankara, Türkiye) with consensus between a radiology resident with 3 years of training and a radiologist with 10 years of experience in abdominal radiology, both of whom were blinded to the clinical data of the patients.

### Measured Factors

**Fig. 1:**
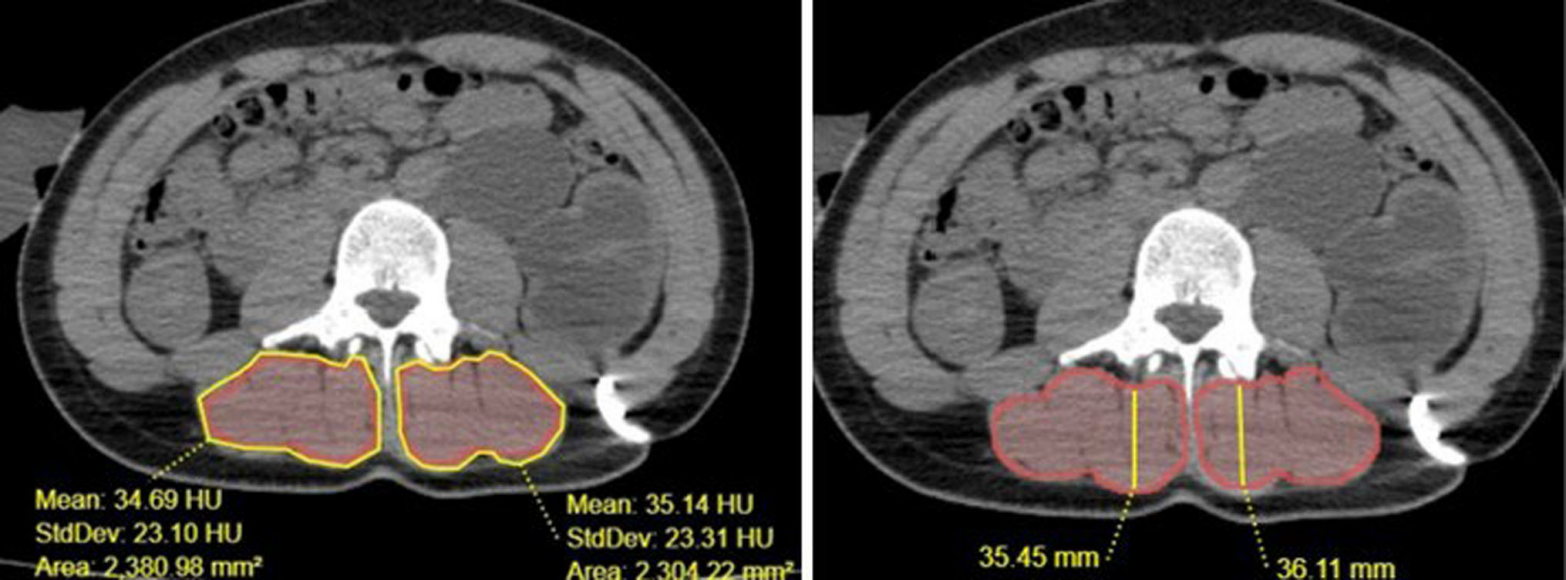
Measurement of the paravertebral muscle area and thickness at the skin entry point of catheter insertion

**Fig. 2:**
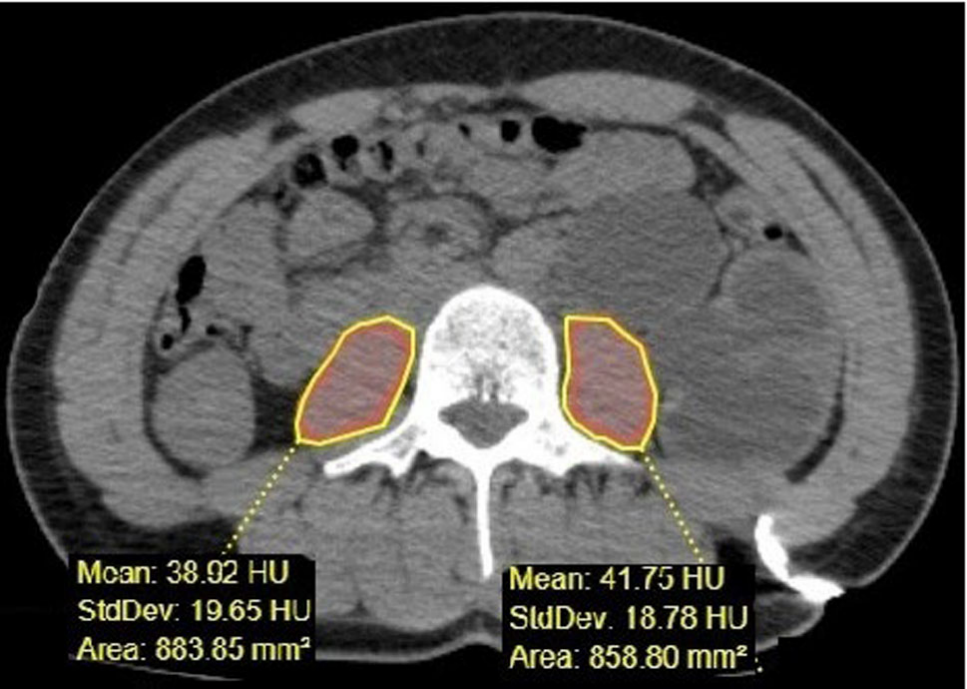
Measurement of the total bilateral psoas muscle area at the catheter insertion point

**Fig. 3:**
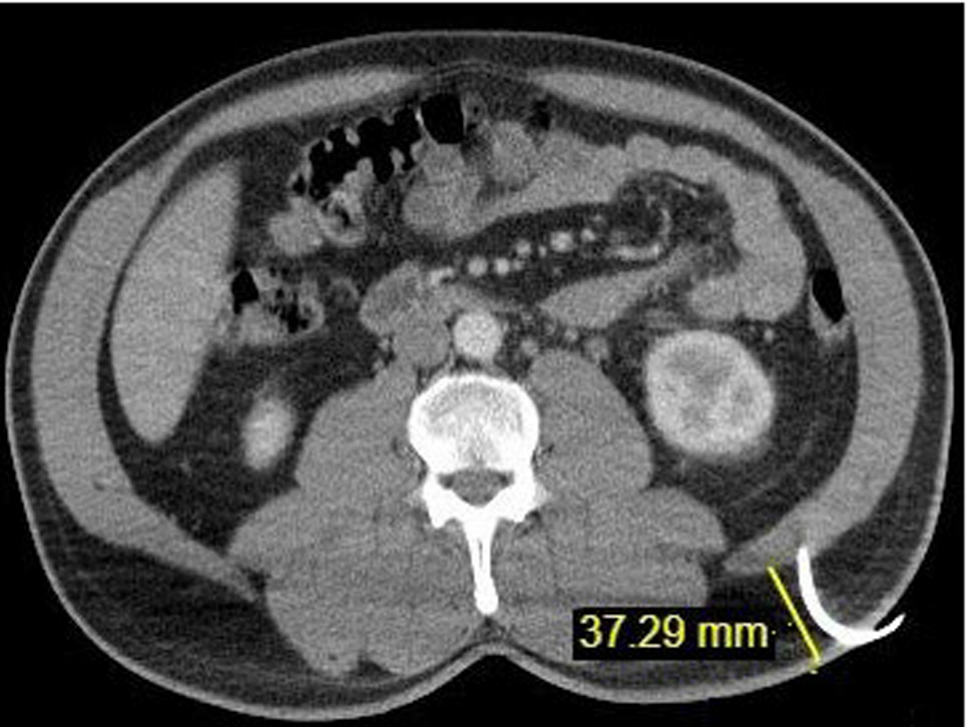
Measurement of subcutaneous fat tissue thickness at the thickest anterior point along the path where the catheter traverses from the skin to the kidney

The total bilateral paravertebral muscle area at the level of nephrostomy catheter passage through the kidney was manually measured by drawing a region of interest (ROI). Additionally, the thickness of the paravertebral muscles at the same level was measured (Fig. 1). The total bilateral psoas muscle area (PMA) at the level of nephrostomy catheter penetration into the kidney was also manually measured by drawing a region of interest (Fig. 2) [17, 18]. The thickness of the subcutaneous fat tissue was measured at its thickest anterior point along the path where the catheter traversed from the skin to the kidney (Fig. 3).

**Fig. 4:**
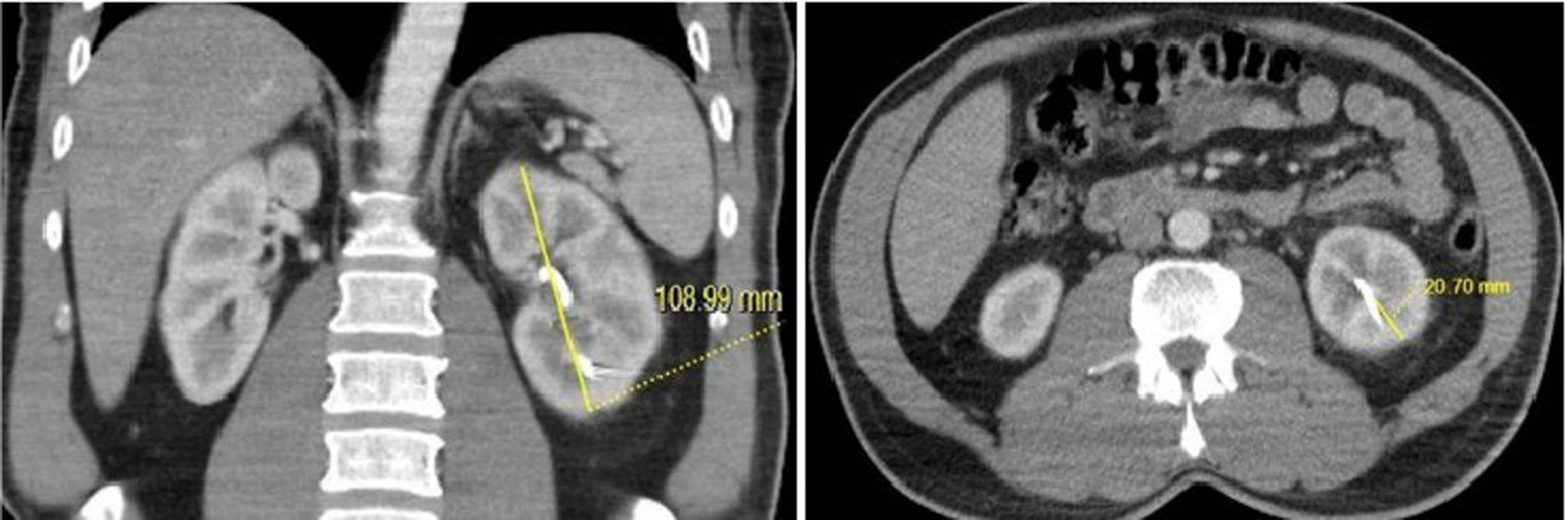
Measurement of kidney size on the coronal plane and parenchymal thickness at the lower pole where the nephrostomy catheter was inserted

**Fig. 5:**
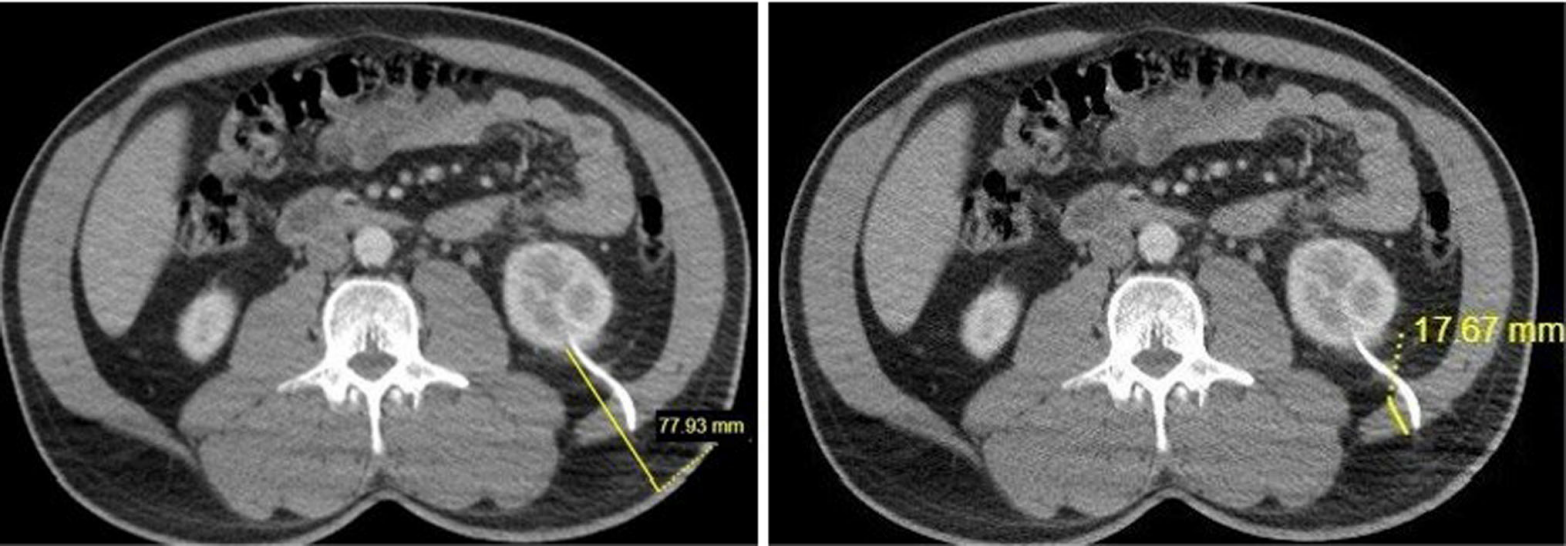
Measurement of the distance from the renal cortex to the skin along the nephrostomy catheter path and the thickness of the posterolateral abdominal wall muscle in the nephrostomy tract

**Fig. 6:**
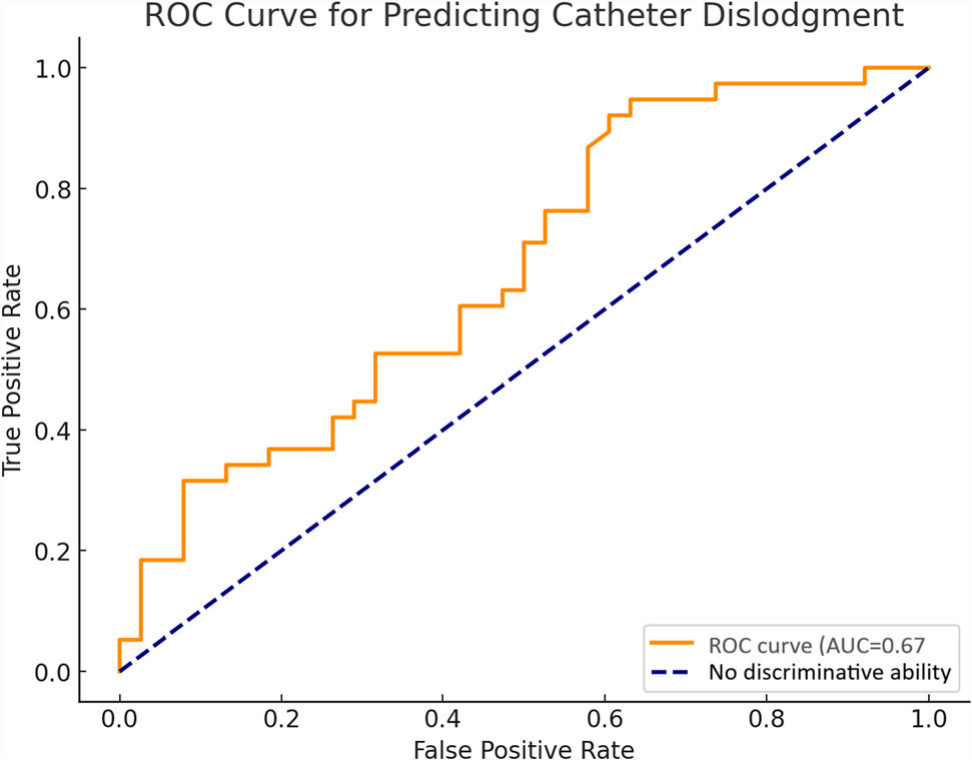
ROC analysis of cortex-to-skin distance

The size of the kidney on the coronal plane where the nephrostomy was placed and the parenchymal thickness at the lower pole where the nephrostomy catheter was inserted were determined for all patients (Fig. 4). The distance from the renal cortex to the skin along the path of the nephrostomy catheter was also measured (Fig. 5). Additionally, the thickness of the posterior lateral abdominal wall muscle in the nephrostomy tract was measured (Fig. 6).

Factors affecting the overall body composition such as the total paravertebral muscle area, BMI (body mass index) subcutaneous fat tissue thickness, and total PMA were analyzed. Additionally, the factors affecting the tissues traversed by the catheter, such as kidney size, renal parenchymal thickness, posterior lateral abdominal wall muscle thickness at the catheter insertion site, the number of repeated nephrostomy procedures, and the cortex-to-skin distance along the catheter path, were also analyzed and compared between the two groups.

Data on blood urea nitrogen (BUN), creatinine (Cr), C-reactive protein (CRP), and white blood cell (WBC) levels at the time of nephrostomy or within 10 days post-procedure were retrospectively collected from the hospital information system and PACS. Hydronephrosis at the time of the procedure, right or left sidedness of the nephrostomy, the specific indication for nephrostomy, presence of malignancy, emergency or elective nature of the procedure (urosepsis patients underwent emergency procedures; elective procedures for other cases), patient age, gender, the number of days until catheter dislodgement, the number of previous nephrostomy procedures, fluoroscopy duration during the procedure, and total air kerma levels were also considered.

### Statistical Analyses

Statistical analysis was performed using SPSS for Windows version 18.0 (SPSS Inc., Chicago, IL, USA). The normality of data distribution was assessed through visual methods (histograms and probability plots) and analytical methods (Shapiro–Wilk test). Descriptive statistics for numerical data were reported as mean ± standard deviation or median (interquartile range), as appropriate. Categorical variables were summarized as frequencies and percentages.

For comparisons, the independent samples t-test was applied to normally distributed numerical data, while the Mann–Whitney U test was used for non-normally distributed data. Categorical variables were compared using the Pearson chi-square test.

The diagnostic performance of cortex-to-skin distance in predicting nephrostomy catheter dislodgement (NCD) was evaluated using Receiver Operating Characteristic (ROC) curve analysis. The area under the curve (AUC), optimal threshold, sensitivity, specificity, and positive predictive value (PPV) were calculated. The optimal threshold was determined using Youden’s Index, which maximizes the sum of sensitivity and specificity. A *p* value < 0.05 was considered statistically significant.

## Results

### Patient Demographics and Characteristics

Among 742 PCN procedures, the prevalence of catheter dislodgment was 5.1% (38/742). In the dislodged group, 68.4% were male compared to 44.7% in controls (*p* = 0.159). Other characteristics, such as hydronephrosis and the number of catheter placements, were not significantly different (*p* = 0.986 and *p* = 0.472). The median time to dislodgment was 35.5 days. Placement on the right side (55.3% dislodged vs. 57.9% controls, *p* = 1.0) and fluoroscopy times (*p* = 0.093) were comparable between groups. Malignancy was present in 57.9% of dislodged cases and 68.4% of controls (*p* = 0.476). BMI was not significantly different (24 vs. 27.5, *p* = 0.166) (Table 1).

**Table 1:** Demographics, disease, and laboratory parameters between patients with and without catheter dislodgement

Variables	Total group	Dislodged catheter group (n = 38)	Non-dislodged catheter group (n = 38)	*p* value
Gender/n (%)				
Female	33 (43.4)	12 (31.6)	21 (55.3)	0.064^a^
Male	43 (56.6)	26 (68.4)	17 (44.7)	
Age/mean ± SD	59.68 ± 13.96	64.42 ± 14.75	54.94 ± 11.46	0.336^b^
Hydronephrosis level/median (IQR)	3 (2–3)	3 (2–3)	3 (2–3)	0.986^b^
Number of catheter placements/median (IQR)	2 (1.5–2.5)	2 (1.5–2.5)	2 (1.5–2.5)	0.472^b^
Time to catheter dislodgement (days)/median (IQR)	–	35.5 (13.7–67.2)	–	–
Side/n (%)				
Right	28 (36.8)	14 (36.8)	14 (36.8)	
Left	42 (55.3)	21 (55.3)	21 (55.3)	1^a^
Bilateral	6 (7.9)	3 (7.9)	3 (7.9)	
Fluoroscopy time (min)/median (IQR)	1.6 (1.20–3.75)	2.60 (1.29–4.13)	1.35 (1.17–3.36)	0.093^b^
Total air krema/median (IQR)	39.4 (20.88–91.00)	56.45 (29.07–88.85)	29.7 (17.50–91.00)	0.251^b^
Etiology/n (%)				
Bening	28 (36.8)	16 (42.1)	12 (31.6)	0.476^a^
Malignant	48 (63.2)	22 (57.9)	26 (68.4)	
Elective-emergency/n (%)				
Elective	59 (77.6)	31 (81.6)	28 (73.7)	0.582^a^
Emergency	17 (22.4)	7 (18.4)	10 (26.3)	
Urea/median (IQR)	57.35 (31.93–98.97)	59.25 (32.10–94.67)	49.45 (32.15–126.22)	0.897^b^
Creatinine/median (IQR)	1.86 (1.14–3.78)	1.67 (1.17–2.49)	2.13 (1.11–4.79)	0.196^b^
WBC/median (IQR)	8.15 (6.54–11.53)	8.38 (6.25–11.34)	8.13 (6.82–11.41)	0.701^b^
CRP/median (IQR)	57.0 (19.57–123.05)	50.90 (17.48–173.00)	66.25 (22.88–108.75)	0.533^b^
Body mass index/median (IQR)	25.00 (20.00–30.25)	24.00 (19.00–27.75)	27.50 (21.0–31.0)	0.166^b^

^a^Pearson ki-kare testi; ^b^Mann–Whitney U testi

### Diagnosis Distribution

The three most common diagnoses were kidney stones (28.9% total; 36.8% dislodged, 21.1% controls), bladder cancer (25%; 26.3% dislodged, 23.7% controls), and prostate cancer (14.5%; 13.2% dislodged, 15.8% controls) (Table 2).

**Table 2:** Diagnosis distribution of all patients and comparison between dislodged and non-dislodged groups

Diagnosis	Total group (n = 76)	Dislodged catheter group (n = 38)	Non-dislodged catheter group (n = 38)
	n (%)	n (%)	n (%)
Kidney stones	22 (28.9)	14 (36.8)	8 (21.1)
Bladder cancer	19 (25.0)	10 (26.3)	9 (23.7)
Prostate cancer	11 (14.5)	5 (13.2)	6 (15.8)
Cervical cancer	7 (9.2)	3 (7.9)	4 (10.5)
Endometrial cancer	4 (5.3)	1 (2.6)	3 (7.9)
Ureteropelvic junction stenosis	1 (1.3)	1 (2.6)	–
Pyelonephritis	2 (2.6)	1 (2.6)	1 (2.6)
Urothelial carcinoma	1 (1.3)	1 (2.6)	–
Metastasis	6 (7.9)	2 (5.3)	4 (10.5)
Retroperitoneal fibrosis	1 (1.3)	–	1 (2.6)
Endometriosis	2 (2.6)	–	2 (5.3)

### Anatomical and Imaging Parameters

The median subcutaneous fat thickness did not differ significantly between the groups (22.46 mm vs. 32.19 mm, *p* = 0.062). Other parameters, including PMA (*p* = 0.593), paravertebral muscle area (*p* = 0.138), paravertebral muscle thickness (*p* = 0.771), kidney coronal length (*p* = 0.839), lower pole renal parenchymal thickness (*p* = 0.644), and posterolateral muscle thickness (*p* = 0.740), were similar between the two groups. However, the cortex-to-skin distance was significantly shorter in the catheter dislodgment group compared to the controls (*p* = 0.001) (Table 3).

**Table 3:** Comparison of measurement values between dislodged and non-dislodged groups

Parameter	Total Group (n = 76)	Dislodged Catheter Group (n = 38)	Non-Dislodged Catheter Group (n = 38)	*p*
	Mean ± SD	Mean ± SD	Mean ± SD	
	Median (IQR)	Median (IQR)	Median (IQR)	
Subcutaneous fat thickness	28.17 ± 12.68	25.40 ± 10.85	30.95 ± 13.87	0.062^a^
	26.24 (17.69–36.20)	22.46 (17.46–32.70)	32.19 (20.35–38.01)	
Psoas muscle area	1578.67 ± 600.14	1636.20 ± 640.31	1521.15 ± 559.70	0.593^a^
	1407.70 (1144.79–1931.07)	1463.56 (1080.64–2032.13)	1368.47 (1154.92–1873.25)	
Paravertebral muscle area	2813.86 ± 974.48	2299.26 ± 854.15	2866.07 ± 976.20	0.138^a^
	2620.84 (2156.49–3564.04)	2168.09 (1741.99–2612.86)	2736.77 (2181.32–3603.17)	
Paravertebral muscle thickness	51.74 ± 8.35	52.13 ± 9.03	51.34 ± 7.59	0.771^a^
	50.66 (45.21–57.85)	51.41 (46.12–57.47)	49.97 (45.06–58.77)	
Kidney coronal length	113.02 ± 14.76	100.65 ± 22.29	101.15 ± 18.81	0.839^a^
	110.24 (101.04–124.47)	102.51 (86.73–115.21)	96.82 (89.64–111.33)	
Lower pole renal parenchymal thickness	15.29 ± 4.82	15.76 ± 5.35	14.81 ± 4.25	0.644^a^
	14.93 (12.06–18.29)	14.84 (12.44–18.39)	14.93 (11.96–17.70)	
Cortex-to-skin distance	58.39 ± 17.32	53.28 ± 16.64	63.50 ± 15.40	**0.001**^**a**^
	56.79 (46.82–69.52)	51.08 (40.79–66.20)	63.31 (49.86–73.73)	
Posterolateral muscle thickness	12.02 ± 3.3.64	12.29 ± 4.03	11.76 ± 3.24	0.740^a^
	11.77 (9.52–14.21)	12.07 (9.21–14.96)	11.74 (10.04–13.44)	

^a^Mann–Whitney U testi

### Predictive Value of Cortex-to-Skin Distance

ROC analysis revealed an AUC of 0.67 for cortex-to-skin distance, with an optimal threshold of 46.65 mm, sensitivity of 92.1%, specificity of 39.5%, and PPV of 60.3% (Fig. 6).

## Discussion

This study aimed to identify factors influencing unintended nephrostomy catheter dislodgment (NCD) after placement, with cortex-to-skin distance shortening emerging as a key determinant.

Unintended NCD is a frequent complication with a wide incidence range. NCD occurred in 26%, 36%, 53%, and 62% of patients (n = 283) at 6, 12, 24, and 36 months after placement, respectively [19]. The clinical burden is significant, requiring time-intensive re-interventions that increase patient discomfort, radiation exposure, and healthcare costs [8, 14, 20]. NCD is implicated in 52–79% of catheter malfunction cases [4, 9, 21]. Its high frequency and risks underscore the need for effective prevention.

The nephrostomy catheter traverses multiple anatomical layers, including skin, subcutaneous fat, abdominal muscles, retrorenal and perinephric fat, and renal parenchyma, to access the collecting system [22]. This study identified the cortex-to-skin distance as a critical factor. A threshold of 46.65 mm was associated with a significantly elevated dislodgment risk. Shorter distances may reduce stabilizing forces, increasing susceptibility to NCD. Patients above this threshold had a reduced risk, supported by high sensitivity (92.1%).

However, specificity (39.5%), positive predictive value (60.3%), and AUC (0.67) highlight the limitations of this metric alone. High sensitivity aids in identifying at-risk patients, but low specificity results in false positives, potentially leading to unnecessary monitoring or interventions. Thus, cortex-to-skin distance should be interpreted within a broader clinical framework.

The moderate predictive performance (AUC = 0.67) underscores the importance of a multifactorial approach. Including clinical and procedural variables—such as age, sex, comorbidities, body composition metrics, and catheter fixation techniques—may enhance predictive accuracy. Anatomical differences influenced by age and sex, like variations in muscle mass and fat distribution, could affect catheter stability. Comprehensive models incorporating these factors are crucial for more robust risk stratification.

Future studies should focus on integrating anatomical, demographic, and procedural factors into risk models. This could improve understanding of NCD mechanisms and support strategies to enhance patient outcomes. Other parameters, including muscle thickness, subcutaneous fat thickness (*p* = 0.055, borderline significance), and renal parenchymal thickness, were not significantly associated with NCD in this study. However, the cortex-to-skin distance provides essential anatomical insights into catheter stabilization.

Patients with shorter cortex-to-skin distances may benefit from enhanced monitoring or adjunctive fixation techniques, such as hub sutures or securement devices, to mitigate NCD risk. Clinical application of these findings could help optimize catheter stability and improve outcomes.

The role of BMI in catheter dislodgment remains inconclusive, with conflicting evidence in the literature. Some studies associate higher BMI with increased NCD risk, while others report no significant correlation [13, 15]. Our study did not match for BMI in propensity-score adjustments, as doing so might have obscured key anatomical differences, such as muscle composition and other body structure metrics, which are central to our investigation of NCD risk.

Our results contrast with David et al., who reported a higher BMI in dislodgment cases (mean BMI: 39.7 vs. 30.9 in controls) [15]. However, their study used larger catheters (Malecot, Modified Foley) after percutaneous nephrolithotomy (PCNL) with tract dilatation, whereas our study utilized standardized 8F pigtail catheters without prior dilatation. Furthermore, their predominantly obese cohort may have been influenced by biomechanical factors such as flank pannus mobility, which were less relevant in our cohort (mean BMI = 25).

Conversely, our findings align with Navarrete et al., who found no significant association between BMI and NCD in a cohort primarily composed of oncology patients with pigtail and Foley catheters [13]. In contrast, Alam et al. reported BMI-related dislodgment but included patients with malignant ureteral obstruction, prior surgeries, radiation therapy, or infections—factors excluded in our study to minimize confounding [8].

These differences highlight the context-dependent nature of BMI's impact on NCD and emphasize the need for larger, BMI-stratified studies to clarify its role in catheter stability.

Preventive strategies for patients with shorter cortex-to-skin distances include regular catheter checks, use of self-locking catheters, securement devices, and timely replacements [9, 12, 13, 23–26]. Given the relatively low specificity and AUC of cortex-to-skin distance, incorporating additional factors such as patient demographics, comorbidities, and catheter care practices into risk models is essential for better identification of high-risk patients.

### Limitations

This study has several limitations. The retrospective, single-center design and relatively small sample size may restrict the generalizability of our findings. The reliance on age and sex as matching variables, while justifiable due to their availability and clinical relevance, may overlook other potential confounders, such as comorbidities or catheter care practices. Although BMI would have been a useful variable for propensity-score matching, it was not included in our study due to data limitations, which may have affected the balance of unmeasured confounders. Additionally, the inclusion of imaging parameters, such as paravertebral and psoas muscle measurements, was exploratory in nature, and their lack of significance highlights the need for further validation in larger cohorts. Variability in procedural techniques among multiple operators and the absence of data on catheter care practices and patient education levels further limit the study’s scope. Addressing these limitations through prospective, multicenter studies with standardized protocols could provide a more robust understanding of the factors influencing catheter dislodgment.

## Conclusion

This study identified a shorter cortex-to-skin distance as a significant factor associated with unintended NCD. These findings may aid in predicting dislodgment risk, facilitating more proactive and targeted management in clinical practice.

## Abbreviations

AUC: Area under the curve
BUN: Blood urea nitrogen
Cr: Creatinine
CRP: C-reactive protein
IRB: Institutional Review Board
NCD: Nephrostomy catheter dislodgement
PACS: Picture Archiving and Communication System
PCN: Percutaneous nephrostomy
PMA: Psoas muscle area
PPV: Positive predictive value
ROC: Receiver operating characteristic
WBC: White blood cell
